# Analytical Methods Based on the Mass Balance Approach for Purity Evaluation of Tetracycline Hydrochloride

**DOI:** 10.3390/molecules28227568

**Published:** 2023-11-13

**Authors:** Sunyoung Lee, Song-Yee Baek, Ha-Jeong Kwon, Ki Hwan Choi, Jeesoo Han

**Affiliations:** Division of Chemical and Biological Metrology, Korea Research Institute of Standards and Science (KRISS), Deajeon 34113, Republic of Korea; songyee.baek@kriss.re.kr (S.-Y.B.); hajeong.kwon@kriss.re.kr (H.-J.K.); kihwan.choi@kriss.re.kr (K.H.C.); hjs715@kriss.re.kr (J.H.)

**Keywords:** tetracycline hydrochloride, mass balance approach, purity assay, ion chromatography-conductivity detector, head-space gas chromatography mass spectrometry, q-NMR

## Abstract

Analytical methods based on the mass balance approach were developed for the purity evaluation of tetracycline hydrochloride, a representative salt compound used in pure veterinary drug analysis. The purity assignment method was used to quantify individual classes of impurities via independent analytical techniques. The mass fraction of the free base or salt form contained in a high-purity organic compound with a hydrochloride salt can be determined. The chloride content by ion chromatography-conductivity detector (IC-CD) and general classes of impurities, including structurally related impurities by liquid chromatography–ultraviolet (LC-UV) detector, water by Karl Fischer (KF) coulometric titration, residual solvents by headspace sampler gas chromatography/mass spectrometry (HS-GC/MS), and non-volatiles by thermogravimetric analyzer (TGA), were considered to calculate the purity of the mass fraction. The chloride content of the salt compound can be considered the main impurity in the mass fraction of the free base in the salt compound. A purity assay using quantitative nuclear magnetic resonance (q-NMR) as a direct determination method was performed to confirm the results of the mass balance method. The assigned purities of the tetracycline free form and its salt form in mass fraction were (898.80 ± 1.60) mg/g and (972.65 ± 1.58) mg/g, respectively, which are traceable to the international system of units (SI). Thus, the procedure for evaluating the purity of the free base and salt forms in the salt compound is newly demonstrated in this study.

## 1. Introduction

The purity evaluation of a pure organic compound using valid methods is essential to be metrologically traceable to the International System of Units (SI) for chemical measurements. Pure organic substances, as primary standard materials, are crucial in characterizing certified reference materials (CRM) [1,2,3]. The mass balance approach (MBA) has been widely applied to determine the mass fraction of the main components of pure organic material [4,5,6,7,8,9,10,11]. Purity assignment based on an MBA requires various analytical methods to estimate the possible impurities in organic materials. An analytical approach based on chemical properties should be explored to evaluate the primary components and various impurities. Karl Fischer (KF) coulometric titration combined with the oven method is typically applied to determine the water content of a material. Thermogravimetric analysis is used to evaluate the nonvolatile impurity content. Residual organic solvents can be quantified using headspace gas chromatography–mass spectrometry. Because of the diverse characteristics of chemical substances, applying a specific analytical method to separate or detect the main component and its impurities is challenging. A compound’s purity can be determined using chromatographic techniques appropriate for its volatility, polarity, and thermal stability. For thermolabile substances, a liquid chromatography–ultraviolet (LC-UV) detector is usually used to evaluate the main components and structurally related impurities [5,8]. Endosulfan-II, a typical volatile material, was analyzed and previously evaluated using a gas chromatography–flame ionization detector (GC-FID) based on MBA [7]. The purity of materials without chromophores, such as monosaccharides, was evaluated using high-performance anion exchange chromatography with pulsed amperometric detection (HPAEC-PAD) [9]. As described in previous studies, the purity values assigned by MBA can be applied to correct the concentration of the standard solution for the quantification [2,3]. Comprehensive analytical methods for purity evaluation were employed in these studies to obtain reliable and SI-traceable values for salt compounds.

Tetracyclines have been the most widely used antibiotics for humans and animals since their discovery as natural products of actinomycete soil bacteria in the late 1940s [12,13,14]. Tetracycline hydrochloride (TCH), the hydrochloride salt of tetracycline, was introduced in 1953; it has been used to treat various infectious diseases [15,16,17,18]. Drug salt formation is a commonly used process to increase drug solubility during drug development [19]. High-purity TCH, a representative hydrochloride salt form of drug materials, can be employed as the primary reference material and calibrator in analytical measurements, such as monitoring it in foods [20,21]. The concentration of the standard solution can be corrected with well-assigned purity of the standard material and consequently the measured results are more accurate with the measurement traceability. However, determining the purity of the hydrochloride salt compound is critical for developing the principal reference material in applications related to veterinary drug residues. The mass fraction assignment of a salt compound can be the content of a free base or salt, depending on the purpose of use. The free base demonstrates pharmacological and antibiotic activities when the salt compound is dissolved with a hydrochloride salt. Because, as mentioned earlier, salt aids in compound solubility, accurate detection of the free base in the salt compound is critical for drug residue analysis. Hence, the Bureau International des Poids et Mesures (BIPM) conducted the Capacity Building and Knowledge Transfer program for tetracycline purity evaluation, and our laboratory participated as a national metrology institute with established capabilities in pure organic material characterization [22]. This study demonstrates a detailed characterization method for assigning the mass fractions of salt and free forms in high-purity TCH materials.

## 2. Results and Discussion

### 2.1. LC-UV Analysis for Evaluation of Tetracycline and Its Analogues

**Figure 1:**
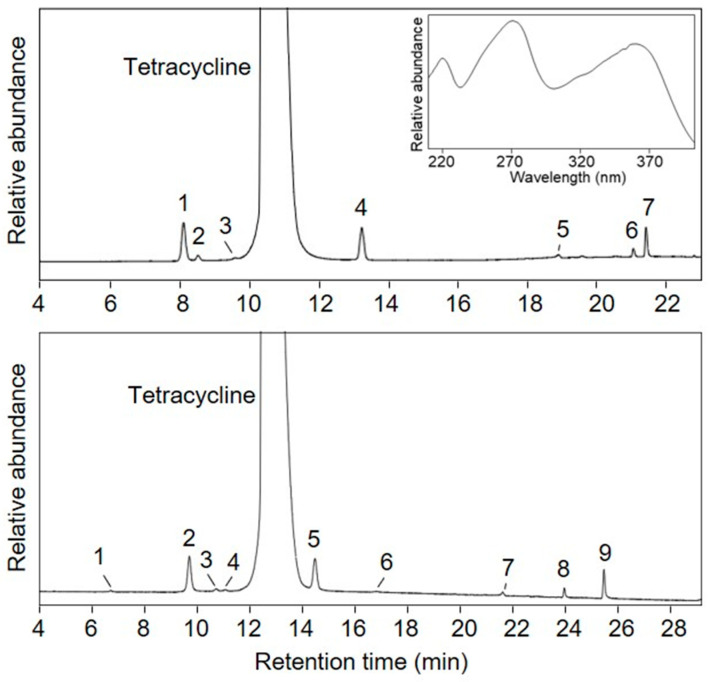
LC-UV chromatogram of tetracycline hydrochloride analyzed by using C8 (top: 1; 4-epitetracycline, 2, 3, and 4; unknown, 5; chlortetracycline, 6; 4-epianhydrotetracycline, 7; anhydrotetracycline) and T3 column (bottom: 1; unknown, 2; 4-epitetracycline, 3; oxytetracycline, 4, 5, and 6; unknown, 7; chlortetracycline, 8; 4-epianhydrotetracycline, 9; anhydrotetracycline).

Tetracycline contains analogs as impurities that are difficult to eliminate during manufacturing because of their similar chemical structures. The main components and structurally related impurities were analyzed using the LC-UV method. The separation conditions using different columns and mobile phase solvents were optimized to resolve possible impurities in the sample. Individual tetracycline analog solutions, including 4-epitetracycline, oxytetracycline, chlortetracycline, 4-epianhydrotetracycline, and anhydrotetracycline, were prepared and analyzed using the LC-UV method with BEH C8 and HSS T3 columns. Figure 1 depicts the LC-UV chromatograms of the tetracycline solution obtained using the two columns. The LC-UV separations demonstrated similar patterns; however, LC-UV analysis using the HSS T3 column could only distinguish oxytetracycline from tetracycline. Other impurities were separated from the main peaks using both columns. The mass fraction of tetracycline was calculated by LC-UV analysis using an HSS T3 column, according to Equation (1).

(1) $$P_{LC-UV}=\frac{A_{TET}}{A_{TET}+\sum_{i}A_{impuritiy,i}}$$

where *P_LC-UV_* is the mass fraction of tetracycline, *A_TET_* is the peak area ratio of tetracycline, and *A_impurity,i_* is the peak area ratio of impurity *i* on the LC chromatogram (at λ_max_) of tetracycline.

Tetracycline was the baseline recovered from the structurally related impurities using the LC-UV method with both columns, as depicted in Figure 1. Several related structural impurities have been identified by LC-UV analysis of commercially available tetracycline analogs, including 4-epitetracycline, oxytetracycline, chlortetracycline, 4-epianhydrotetracycline, and anhydrotetracycline. The mass fraction of tetracycline was assessed by LC-UV analysis using the T3 column. The LC separation using the C8 column contained seven impurities. The T3 column could separate more impurities (nine) than the C8 column, and the T3 column was able to separate oxytetracycline from tetracycline. The purity of tetracycline in the mass fraction was determined from five LC-UV measurements and the average value with the expanded uncertainty at a 95% confidence level was (98.080 ± 0.023)%.

### 2.2. KF Coulometry for Determination of Water Content

The KF coulometric titration was performed using the oven method to determine the sample’s water content. The temperature for the KF oven method is normally set to 20–30 °C below the substance melting point. The initial temperature when the substance turned dark brown and the water contents varied with the measurements was 190 °C. Based on the TGA analysis, a significant mass change of the substance occurred at 140 °C. Therefore, water content was determined to maintain the state and color of the substance. The commercial oven water standard was analyzed at 140 °C to ensure the KF coulometric titration method. The water contents of the samples were determined using Equation (2).

(2) $$P_{water}=\left(ICEQ/10.712-Time\times Drift-Blank\right)/m\times C$$

where *P_water_* is the water content mass fraction in the sample, and *ICEQ* is the total consumed electric charge. *Time* is the measurement time in minutes, *Drift* is the water content using the KF coulometric system before analysis, *Blank* is the average water content in six empty vials, *m* is the sample mass used for KF coulometric titration, and *C* is a constant, 1 × 10^6^.

Six system blanks for the water content determination in the material were analyzed before the sample analysis, ranging from 90 to 100 µg/min. The water content of TCH in mass fraction was calculated from five KF coulometric measurements with the oven method; the average value with the expanded uncertainty at a 95% confidence level was (0.664 ± 0.060)%.

### 2.3. TGA for Determination of Nonvolatile Impurity Content

The nonvolatile impurity content was determined using the difference in weight before and after TGA. The total nonvolatile impurity content in the mass fraction (*P_nonvolatile residue_*) was calculated using Equation (3).

(3) $$P_{nonvolatile\;residue}=\frac{m_{nonvolatile\;residue}}{m_{sample}}$$

where *m_nonvolatile residue_* is the difference in pan mass before and after TGA analysis, and *m_sample_* is the mass of the sample used for TGA.

The thermal program of the TGA was designed to ensure complete oxidative combustion of the substance and to retain nonvolatile residues, such as inorganic materials. However, there was no difference in the mass of the empty pan after TGA during the six TGA measurements. This outcome indicates that nonvolatile residues did not exist in the high-purity TCH.

### 2.4. Headspace-GC/MS for Evaluation of Residual Organic Solvents

**Figure 2:**
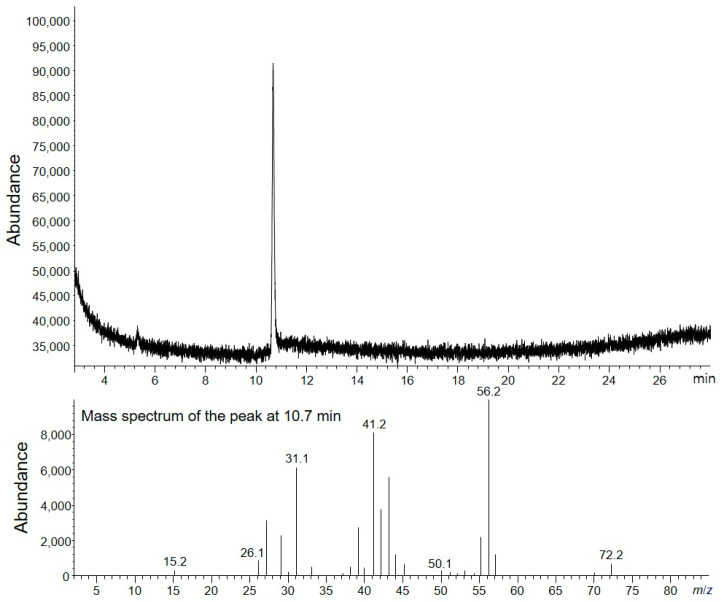
TIC chromatogram of TCH analyzed by HS-GC/MS.

**Figure 3:**
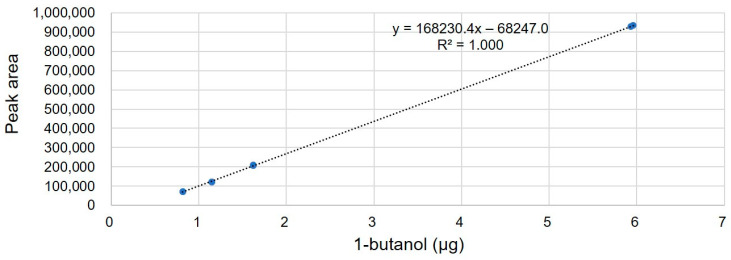
Linear regression plot of 1-butanol in DI water.

Three replicates of TCH (2–3 mg) were prepared as described in the Experimental Section, and HS-GC/MS analysis was performed. The TIC chromatogram of the HS-GC/MS analysis revealed one peak of the residual solvent (Figure 2). Based on the MS spectrum and GC retention time, a peak corresponding to 1-butanol was confirmed. The quantity of 1-butanol in TCH was calculated from four concentration levels of 1-butanol calibration standard solutions. The linearity of the method was investigated in water at four concentration levels in the range 0.5–6 µg, based on a 2–3 mg sample. Two replicates were used for each level. The linear regression plots are depicted in Figure 3. The correlation coefficient (R2) was 0.9999. The residual organic solvents in the mass fraction (*P_volatile_*_*organic solvent*_) were calculated using Equations (4) and (5).

(4) $$P_{volatile\;organic\;solvent}=\frac{{\sum m}_{volatile\;organic,i}}{m_{sample}}$$

(5) $$m_{volatile\;organic}=\frac{A_{volatile\;organic}-y_{intercept}}{Slope}$$

where *m_volatile organic,i_* is the mass of the volatile organic solvent, *m_sample_* is the mass of the sample, *A_volatile organic_* is the peak area of the volatile organic solvent, *y_intercept_* is the intercept of the calibration curve, and Slope is the slope of the calibration curve.

The measurement results of residual solvent in TCH are listed in Table 1. In three replicates, 0.162% of the residual solvent was averagely detected. The standard deviation of residual solvent content in three replicates was 0.007%, which indicates that the TCH sample was homogeneous and the method has reproducibility. The residual solvent content was (0.162 ± 0.151)%, representing the average value of three replicates, and the expanded uncertainty was calculated considering the standard deviation of three replicates and the standard uncertainty of the measurement procedure.

### 2.5. IC-CD for Determination of Chloride Content

**Figure 4:**
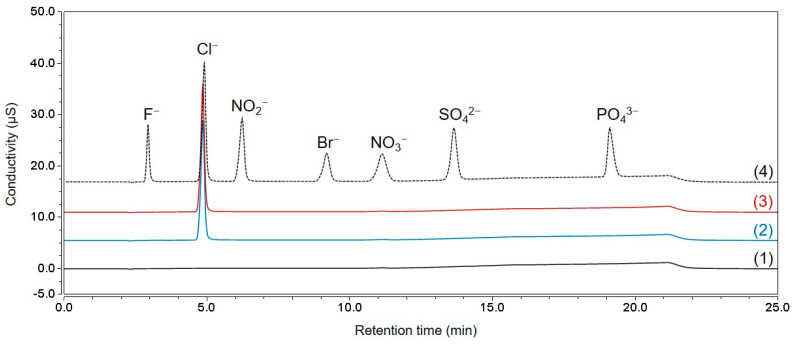
IC-CD chromatograms of blank DW (1), chloride standard solution (10 mg/kg) (2), tetracycline hydrochloride solution (140 mg/kg) (3), and seven anion mixture (4).

Ion chromatography-conductivity detector (IC-CD) was used to analyze chloride in TCH. Figure 4 shows the IC-CD chromatograms of the seven-anion mixture, blank DW, standard chloride solution, and tetracycline samples. The chromatogram demonstrated that other major anions (fluoride, bromide, nitrite, nitrate, phosphate, and sulfate), except chloride, were not discovered in the sample; thus, only the chloride concentration was measured using Equation (6).

(6) $$P_{Cl}=\frac{C_{std}\times A_{sample}}{C_{sample}{\times A}_{std}}$$

where *P_Cl_* is the mass fraction of chloride in the sample, *C_std_* and *C_sample_* are the concentrations of chloride in the selected standard solution and TCH sample, respectively, and *A_std_* and *A_sample_* are the average peak areas obtained by analyzing the standard solution and sample, respectively. Among the four standard solutions, the one with an intermediate response factor value was selected as the calibration solution.

The chloride content was measured at 0.0732 g/g (7.32%); thus, the hydrochloride content can be calculated to be 0.0753 g/g (7.53%), assuming that the moles of chloride and protons were the same. The relative systemic uncertainty was 0.219% by combining the uncertainty of KRISS CRM, the variations in response factors from four standard solutions, and variations in the repeated analysis of a standard solution. The random uncertainty was calculated as 0.073% from the measurement results of four TCH samples. When the systemic and random uncertainty were combined, the relative standard uncertainty was 0.231%.

The presence of counter ions in charged organic compounds complicates the purity assessment of high-purity salt compounds, particularly when the molar numbers of the two ions do not match. This study’s aim was to perform a purity assessment of tetracycline, a zwitterion that combines protons and chlorides. Whenever the moles of chloride exceed the moles of the tetracycline-free base, the excess chloride may be bound to other cations, and vice versa, and the presence of excess tetracycline as an inner salt form or other anions should be considered.

### 2.6. NMR for Purity Evaluation of TCH

**Figure 5:**
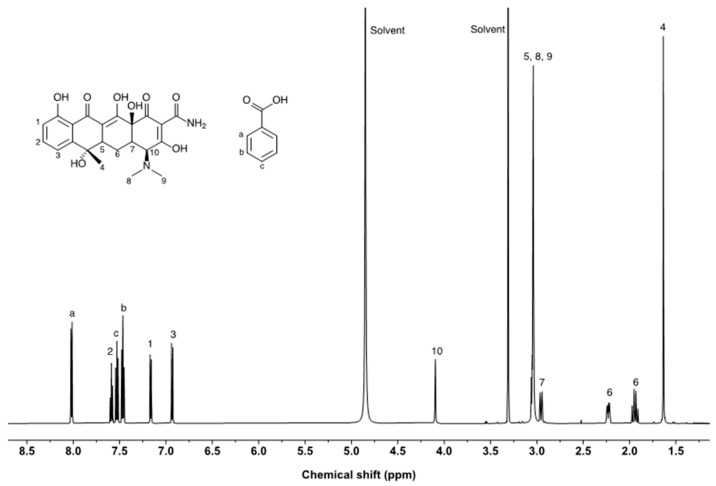
H NMR spectrum of TCH. 1–10, and a–c correspond to the proton positions of tetracycline and benzoic acid, respectively.

The purity of tetracycline was assessed via qNMR measurements using benzoic acid as an internal standard. The NMR resonances of tetracycline and benzoic acid are well separated (Figure 5). However, the 1H NMR spectrum of tetracycline exhibited multiple overlapping NMR resonances in the upfield (0–3.5 ppm). NMR peaks from the protons at positions 1, 2, 3, and 10 were considered candidates for purity assessment. The epimerization of tetracycline was observed at position 10, and the NMR peak area ratio of the proton decreased when the sample solution was reanalyzed after 3 days. Because the resonances from positions 1 and 2 were relatively similar to the resonance of benzoic acid, those from position 3 were selected for purity assessment. Three independent solutions of tetracycline were prepared and measured in triplicate for each solution. The purities of the tetracycline-free base and salt forms were calculated using Equation (7).

(7) $$P_{a}=\frac{I_{a}N_{s}M_{a}W_{s}}{I_{s}N_{a}M_{s}W_{a}}P_{s}$$

where *P* is the purity, *I* is the integrated peak area, *N* is the number of ^1^H atoms contributing to the signal area, *M* is the molecular weight, *W* is the weight, and the subscripts “*a*” and “*s*” refer to the analyte and the internal standard, respectively. For the purity assessment of the tetracycline-free base and salt forms, molecular weights of 444.4 and 480.9 were used, respectively. The purities and expanded uncertainties of the tetracycline-free base and salt form were (909.3 ± 2.2) mg/g and (983.9 ± 2.4) mg/g, respectively.

The purity assessed using qNMR was higher than that determined using the balance method. In qNMR measurements, the purity can be overestimated because of the peak overlap between tetracycline and impurities. Small peaks were observed near the tetracycline peaks due to protons from impurities that have a similar structure to tetracycline. LC-UV analysis demonstrated that structure-related impurities were present in tetracycline. The results indicate that the confirmation of peak overlap is critical for accurate purity determination when qNMR is used to assess the purity of high-purity chemicals. In addition to tetracycline, butanol resonances were observed at 1.3–1.5 ppm. The butanol content of the tetracycline reagent was 0.17%, which is consistent with the results of headspace GC/MS measurements.

### 2.7. Purity Assignments of Tetracycline Free Form and Its Salt Form

The final mass-fraction assignments of the material free base and salt forms were calculated using the quantification results of the above analytical techniques. Equation (8) was used to determine the purity of the free base and salt forms in the high-purity TCH material.

(8) $$P=(1-\sum P_{impurity})\times P_{LC-UV}$$

The assigned purities of the tetracycline free form and its salt form in mass fraction with the expanded uncertainty at a 95% confidence level were (898.80 ± 1.60) mg/g and (972.65 ± 1.58) mg/g, respectively. A summary of the measured results, including the associated uncertainties from the purity evaluation, is presented in Table 2. The mass fraction of the tetracycline-free base was determined using Equation (8). Quantification of Impurities. Chloride content was considered a primary impurity for calculating the mass fraction of the free base.

For the purity assignment of the salt form, the measurement results from LC-UV, TGA, KFT, and HS-GC/MS (except for IC-CD) were combined. Expected chloride contents are 7.17% based on the final calculated purity (97.27%) of TCH, whereas it is 7.31% based on the purity excluding LC-UV results (99.17%), which was in agreement with the measured value (7.32 ± 0.17)% within the uncertainty range. Therefore, we concluded that no other anions existed in the high purity sample and the final purity for the salt form was calculated under the presumption that tetracycline and its analogues are bound to hydrochloride salt.

## 3. Experimental

### 3.1. Materials and Chemicals

**Figure 6:**
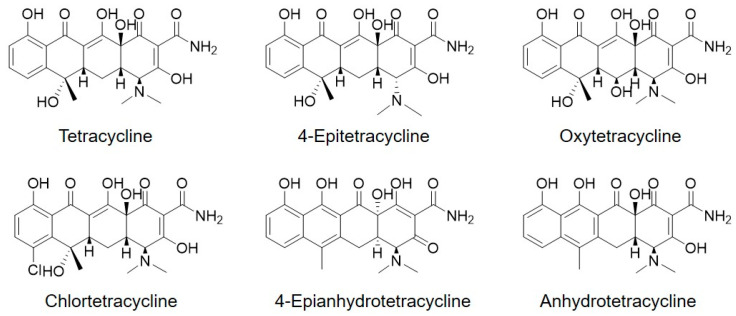
Chemical structures of tetracycline and its analogues.

Two bottles of 500 mg TCH were provided by the BIPM Capacity Building and the Knowledge Transfer (CBKT) Program-Metrology of Safe Food and Feed. Tetracycline analogs, including 4-epitetracycline, oxytetracycline, chlortetracycline, 4-epianhydrotetracycline, and anhydrotetracycline, (Figure 6) in hydrochloride salt form were purchased from Sigma-Aldrich (St. Louis, MO, USA). Acetonitrile and formic acid were obtained from Sigma-Aldrich. Hydranal Coulomat AG was used as the KF titration solvent and was obtained from Merck (Darmstadt, Germany). Methanol-d4 was purchased from Euriso-top (D > 99.80 atom %, Saint-Aubin, France). Benzoic acid (PS1) was obtained from the National Institute of Standards and Technology (Gaithersburg, MD, USA).

### 3.2. LC-UV Analysis

An Acquity UPLC with a PDA detector (Waters, Milford, MA, USA) was used to evaluate structurally related impurities. The LC-UV method has an advantage of wide dynamic range and can be used for the determination of organic substances with chromophore [8]. TCH and its analogs were analyzed by an Acquity UPLC BEH C8 column (100 mm length, 1.7 µm particle size, 2.1 mm i.d.; Waters) and HSS T3 column (100 mm length, 1.8 µm particle size, 2.1 mm i.d.; Waters). The separation conditions using each column were optimized to separate tetracycline (TET) and its analogs from each other. The temperatures of the column and the sampler were set to 30 °C and 5 °C, respectively. LC-UV analysis was performed with a mobile phase containing 0.1% formic acid in water and methanol (solvents A and B, respectively) on a BEH C8 column. LC separation with an HSS T3 column was also performed using water and acetonitrile mixture solvents (95:5 for solvent A, 5:95 for solvent B by volume) with 0.2% formic acid. TCH solutions in water were prepared at concentrations ranging from 50 to 250 mg/kg to investigate the UV response factors. The limit of detection (LoD) of the LC-UV was 0.015 mg/kg (S/N = 3) for TET and the limit of quantitation (LoQ) was 0.05 mg/kg (S/N = 10). Ten microliters of the sample were injected at a flow rate of 0.2 mL/min. The LC-UV chromatogram at 270 nm demonstrated maximum UV absorbance for TCH.

### 3.3. Coulometric KF Titration

A coulometric KF titrator (Titrator Compact C30; Mettler Toledo, Columbus, OH, USA) was used with an oven sample changer. The system was validated using SRM 2890 containing (47.3 ± 1.0) mg/g water and Hydranal oven water standard containing (50.8 ± 0.4) mg/g water. The LoD of the KF titration was 10 µg (S/N = 3) water per sample. Ultra-high pure nitrogen gas (>99.9999%) was used as the carrier gas at a 50 mL/min flow rate. The KF titration system was operated in a glove box under a nitrogen atmosphere to minimize external moisture effects. The maximum drift was set to 15 µg/min, and the mixing time was 600 s. The oven temperature was 140 °C, which is below TCH’s melting point. An external analytical balance (XP205, Mettler Toledo) was used to measure the mass of the samples. Before sample analysis, the water content in six empty vials was measured, and the mean value for the vials was used as the system blank for the water determination of the sample.

### 3.4. TGA

A thermogravimetric analyzer (TGA/DSC1, Mettler Toledo) was used to evaluate the nonvolatile impurity content. Approximately 3 mg of the sample was weighed in a 70 µL alumina pan using an external ultra-micro balance (XP2U, Mettler Toledo). The oven chamber temperature was increased from 25 °C to 600 °C at a rate of 10 °C /min with a nitrogen flow of 50 mL/min and maintained for 300 min with an airflow of 50 mL/min. Before sample analysis, the TGA program was run three times with an empty sample pan to remove residual impurities.

### 3.5. Headspace-Gas Chromatography/Mass Spectrometry

Headspace sampler gas chromatography/mass spectrometry (HS-GC/MS) was used for the residual solvent analysis. The experiments were performed using an Agilent 6890N/5973 gas chromatograph/mass spectrometer (GC/MS) equipped with an Agilent 7697A headspace sampler. A DB-624 GC capillary column (30 m length, 0.32 mm inner diameter I.D., 1.8 µm film thickness) from Agilent Technologies (Santa Clara, CA, USA) was utilized for GC. The inlet temperature was 200 °C and the split ratio was 10 to 1. Helium (99.999%) was used as the carrier gas at a 1.5 mL/min flow rate. The oven’s initial temperature of 40 °C was maintained for 10 min. It was then raised at a rate of 10 °C /min to 200 °C and sustained for 2 min. The transfer line temperature was 260 °C. For MS, electron impact ionization was performed at 70 eV and 230 °C, with a quadrupole temperature of 150 °C. An MS was set to scan a mass range of 10–300 amu. The equilibration, sample loop, and transfer line temperatures for HS were 100 °C, 110 °C, and 120 °C, respectively. The vial equilibration time was 30 min.

A microbalance (UMX2, maximum capacity = 2.1 g, readability = 0.1 µg; Mettler Toledo, Switzerland) and an analytical balance (XP206, maximum capacity = 200 g, readability = 10 µg; Mettler Toledo, Greifensee, Switzerland) were used for weighing. The 2–3 mg tetracycline hydrochloride was weighed into a Pt pan and placed into a 10 mL headspace vial (Agilent Technologies, Santa Clara, CA, USA). The vial was subsequently charged with 1 mL of diluent (deionized (DI) water). DI water was obtained using a Milli-Q water purification system (Millipore Corporation, Bedford, MA, USA). The vials were immediately sealed with an aluminum crimp cap having a PTFE/silicon septum and mixed/sonicated thoroughly until the tetracycline hydrochloride was dissolved. The prepared samples were preliminarily analyzed to confirm the types and expected amounts of residual solvent. MS spectra and GC retention duration were employed to identify the types of residual solvents observed.

Preparation of external calibration standard solutions for preliminarily detected residual solvents: The standard solutions were prepared using a high-purity solvent. Sigma-Aldrich (St. Louis, MO, USA) provided 1-butanol with a purity higher than 99.9%. The standard solution and diluent were added such that the sum of the standard solution and diluent was 1 mL. Using the peak area of the solvent in the sample solution and the external calibration standard solutions, the amount of each solvent used to prepare the standard solution, the weighed amount of the sample, and the content of each residual solvent in the sample were calculated. The LoD of the HS-GC/MS was 0.3 µg (S/N = 3) and the LoQ was 1 µg (S/N = 10). A blank was prepared using 1 mL of deionized (DI) water, the same as when the sample was dissolved.

### 3.6. IC-CD Analysis

IC-CD analysis of chloride and other anions in tetracycline hydrochloride was performed using an ICS-5000 series instrument (Thermo Fisher Scientific, Waltham, MA, USA) comprising a dual pump, eluent generator, column compartment, and autosampler. Chromeleon 7 software was used for the data analysis. The eluent generator consisted of an EGC III potassium-hydroxide eluent generator cartridge, a CR-ATC continuously regenerated anion trap column, and a CRD200 carbonate removal device. Separation was performed on an IonPac AS18 analytical column (250 mm × 2 mm) protected by an IonPac AG18 column (50 mm × 2 mm) with a KOH gradient elution at a 0.2 mL/min flow rate. The KOH concentration in the gradient program is as follows: isocratic elution at 15 mM for 8.0 min, linear gradient elution from 15 mM to 45 mM for 10 min, linear gradient elution from 45 mM to 15 mM for 0.1 min, and finally, reconditioning of the column with 15% for 6.9 min. The column oven temperature was set to 30 °C, and the injection volume was 10 µL. The anion electrolytically regenerated suppressor (2 mm; AERS) was operated in auto-suppression cycle mode, and the suppressor current was 39 mA.

Four chloride standard solutions with chloride concentrations of 10 mg/kg were prepared by gravimetrically diluting KRISS CRM 105-04-002 (KRISS, Deajeon, Republic of Korea) with certified values and an expanded uncertainty of 1001.0 ± 1.2 mg/kg. Four tetracycline hydrochloride samples were gravimetrically prepared at 140 mg/kg in deionized water to obtain the same chloride concentration as the standard solutions. The standard solutions, sample solutions, and blank deionized water were analyzed in five replicates per sample without further sample preparation. We also confirmed whether major anions other than chloride were present in the sample by obtaining chromatograms of seven anion standard mixtures (Dionex) consisting of fluoride, chloride, bromide, nitrite, nitrate, phosphate, and sulfate.

### 3.7. NMR

Three sample solutions were prepared independently for the qNMR experiments. Tetracycline and benzoic acid were weighed using an XP2U ultramicrobalance (Mettler Toledo) and combined in a glass vial. The analyte and internal standard were dissolved in methanol-*d*_4_ NMR measurements, and the solution was transferred into NMR tubes immediately before NMR measurement. qNMR experiments were performed using a VNS-600 (Varian Inc., Palo Alto, CA, USA) NMR spectrometer operating at 600 MHz. The NMR spectrometer was equipped with a 5 mm PFG Dual Broadband 600 NB probe operated by VnmrJ v3.2A software. The 1H NMR spectra of tetracycline were obtained by acquiring 32 scans of 64 K data points with a spectral width of 9615.4 Hz, acquisition time of 3.408 s, relaxation delay time of 60 s, and a flip angle of 90°. The spectra were processed using the MestreNova v12.0.2 software (Mestrelab Research, Santiago de Compostela, Spain).

### 3.8. Uncertainty Evaluation

The standard uncertainty of the impurity content obtained from multiple measurements of independent techniques was determined using in-house protocols according to the Guide to the Expression of Uncertainty in Measurement; detailed descriptions are provided elsewhere [7,8,9,23]. The uncertainty of the purity value in the mass fraction (u_purity_) of the material was evaluated using Equation (9).

(9) $$u_{purity}=\sqrt{u_{impurities}^{2}+u_{LC-UV}^{2}}$$

where u_impurities_ is the uncertainty associated with the impurity measurements, including KF titration, TGA, and HS-GC/MS (including IC-CD for the purity of the free base), and u_LC-UV_ is the uncertainty associated with the LC-UV measurement.

## 4. Conclusions

As the primary measurement standard, the SI-traceable value assignment of pure organic compounds is the ultimate source of meteorological traceability in the analytical measurement of a material in a particular matrix. Valid purity determination by the mass balance approach is required to provide reliability and traceability of the results for sequential chemical measurements. We described a detailed evaluation of the purity of TCH as a representative salt compound. Based on the MBA, the purity of the mass fraction was examined by combining various analytical measurement results obtained from LC-UV, KF coulometry, TGA, headspace-GC/MS, and IC-CD analyses. The analytical methods described for the purity assignment of the tetracycline-free form and its salt form in the mass fraction can be applied to other salt-form compounds used as the primary standard and calibrator.

## Figures and Tables

**Table 1:** Residual solvent contents in the three replicates of TCH.

	Sample Size (mg)	Contents of 1-Butanol		Standard Unc. (%) *	DoF *
		Absolute (μg)	Relative (%)		
Replicate1	2.415	3.77	0.156%	0.072%	8
Replicate2	2.465	4.21	0.171%	0.070%	8
Replicate3	2.282	3.66	0.160%	0.076%	8

* Standard uncertainty and degree of freedom (DoF) of each replicate were evaluated based on the KRISS protocol (this value represents the uncertainty from the measurement procedure, i.e., the calibration curve and detection limit of the representative solvents).

**Table 2:** Summary of the mass fractions and the measurement uncertainties for purity evaluation.

	Mass Fraction (mg/g)	Combined Standard Uncertainty (mg/g)	Coverage Factor ^a^	Expanded Uncertainty (mg/g)
Related structure impurities	19.20	0.08	2.78	0.23
Water	6.64	0.27	2.78	0.74
Nonvolatiles	0.05	0.04	2.57	0.10
Residual organic solvents	1.62	0.73	2.06	1.51
Chloride ion	73.23	0.17	3.18	6.98
Tetracycline (free from)	898.80	0.78	2.05	1.60
Tetracycline hydrochloride (salt form)	972.65	0.77	2.05	1.58

^a^ The coverage factor is *k* with a level of confidence of 95%.

## Data Availability

Data are contained within the article.
